# How are countries dealing with their current cardio-vascular disease burden? A snapshot from the WHO Eastern Mediterranean Region (EMR)

**DOI:** 10.21542/gcsp.2018.1

**Published:** 2018-03-14

**Authors:** Asmus Hammerich

**Affiliations:** Director, Noncommunicable Diseases & Mental Health (NMH) WHO Eastern Mediterranean Regional Office (EMRO) Abdel-Razak El-Sanhouri Street P.O. Box 7608, Nasr City, Cairo 11371, Egypt

## Abstract

In recent years, a number of global commitments have been made in the area of noncommunicable diseases (NCD). These include the *UN NCD Political Declaration* in 2011, and the *UN Comprehensive Review on NCDs and Outcome Document* in 2014. Nine global targets have been agreed in the area of NCDs, and NCDs have been addressed in the Sustainable Development Goals (SDG). Another UN high-level meeting will take place in September 2018 to assess country progress across the globe.

At the regional level, a number of initiates have taken place to deliver on these global commitments. One of the guiding documents is the *Regional Framework for Action on Noncommunicable Diseases*. This framework was endorsed at the WHO EM Regional Committee in 2012, and includes 17 strategic interventions and 10 monitoring indicators, covering the areas of NCD governance, prevention, surveillance and healthcare.

Progress is being monitored on an annual basis through the development of country progress factsheets and biennial WHO Country Capacity Survey on NCDs. To date however, progress has been insufficient and uneven. Moreover, is has been slowest in the areas of planning and surveillance, and tobacco control.

No uniform approach or model exists for all EMR countries, but a number of countries have advanced their national NCD agenda through original and innovative initiatives.

Perceived challenges include the uneven progress and needs across the WHO EM region, humanitarian emergencies and political instability, vertical approaches, a lack of human and financial resources and other health systems weaknesses.

Opportunities however exist through the global SDG and universal health coverage (UHC) agendas offering an opportunity to revisit essential health services package until 2030.

Overall, there has been political commitment to NCD governance, as evidenced by the EM Regional Committee’s endorsement of the regional framework for action. However, despite the clear roadmap, progress has been slow and scattered, differing vastly by country and by topic.

We recommend that countries urgently scale up their efforts in all four areas of the EM Regional Framework of Action to be able to achieve their national and international targets.

## Current WHO EMR country status and progress of noncommunicable diseases (NCD) and cardio-vascular diseases (CVD) prevention and control

In recent years, a number of global commitments have been made in the area of noncommunicable diseases. These include the *UN NCD Political Declaration* in 2011^1^, and the *UN Comprehensive Review on NCD* and *Outcome Document* in 2014^2^. Nine global targets have been agreed in the area of NCD, and NCD have been addressed in the Sustainable Development Goals (SDG)^3^. The third UN high-level meeting will take place in September 2018, and thus the Eastern Mediterranean Region (EMR) Member States (Figure 2) are proactively working towards achieving the targets.

**Figure 1.**
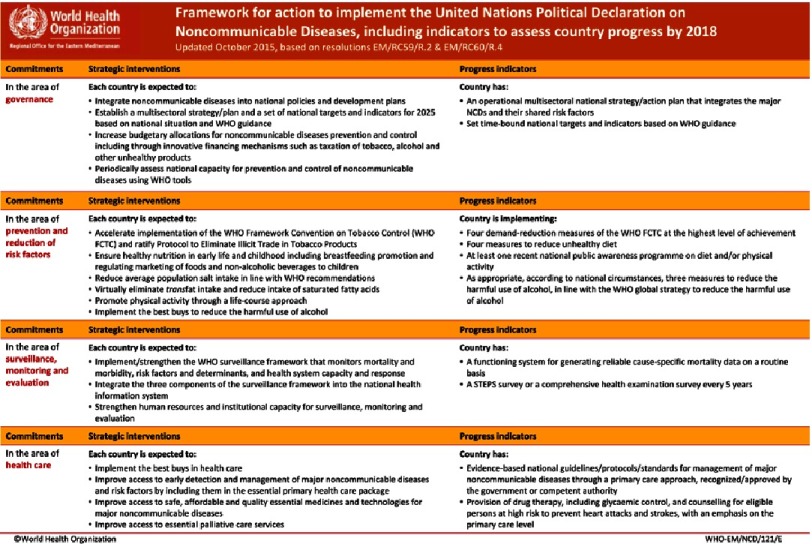
Regional Framework for Action on Noncommunicable Diseases.

At the regional level, a number of initiatives have been taken to deliver on these global commitments. One of the guiding documents is the following *Regional Framework for Action on Noncommunicable Diseases*^4^. This framework was endorsed at the WHO EM Regional Committee in 2012, and includes 17 strategic interventions and 10 monitoring indicators, covering the areas of NCD governance, prevention, surveillance and healthcare (Figure 1).

**Figure 2.**
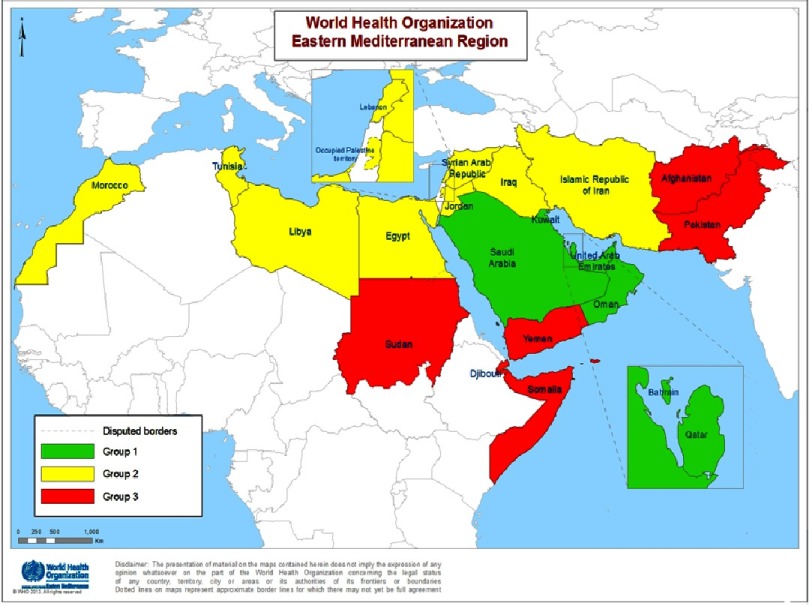
Member States of the WHO Eastern Mediterranean Region, color-coded by population health outcomes, health system performance and level of health expenditure in 2017. (Group 1 comprises countries where socioeconomic and health development has progressed considerably over the past decades. Group 2 comprises largely middle-income countries which have developed extensive public health infrastructure but face resource constraints. Group 3 comprises countries which face constraints in improving population health outcomes as a result of lack of resources, political instability and other complex development challenges).

Progress is being monitored on an annual basis through the development of country progress factsheets on NCDs. The factsheets provide an update for each of the 22 countries, on whether they are fully implementing, partially implementing or not implementing each of the progress 17 sub-indicators. Such information is based on best available evidence, including the data presented in the biennial global WHO Country Capacity Survey on NCDs.

To date, only eight countries in the region are fully achieving six or more of the sub-indicators; the remaining 14 countries are achieving less than one-third of the sub-indicators. Progress is slowest in the areas of planning and surveillance, and tobacco control. Therefore the country progress sheets also provide recommendations to each of the 22 countries, on the work elements they should focus on (Source: WHO NCD Global Progress Monitor 2017^5^).

For monitoring national NCD response, capacity for NCD or CVD management has been assessed in terms of the existence of operational multi-sectoral national strategies or action plans integrating the major NCDs and their shared risk factors, evidence-based national guidelines, protocols or other standards for the management and referral of major NCD (recognized or approved by government or competent authorities), the availability of nine essential NCD medicines at the primary care level, and the provision of drug therapy, including glycemic control and counselling for eligible persons at high risk to prevent heart attacks and stroke^5^.

## Experiences for CVD prevention and control in the WHO EMR

No uniform approach or model exists for all EMR countries. Longstanding vertical programs on hypertension and diabetes mellitus with country tools are in place, but limited integration and focus on total CVD risk, monitoring of health system performance, uptake and sustainable use of WHO *package of essential NCD interventions for primary health care* (PEN) persist. Also, a ‘health system lens’ to identify and address key bottlenecks is missing in many countries. However there appears to be a renewed interest in the launch of Global HEARTS initiative. Some encouraging EMR country experiences include Palestine, Oman, Bahrain (WHO PEN protocol implementation) and Iran (‘IraPEN’ pilot implementation through community health workers (Community health workers (CHW) or ‘bevharzes’) and the Iran CVD-SUPPORT Trial).

The example of IraPEN has a number of outstanding features such as a well-designed package of interventions covering the four main NCD and their related risk factors adapted from WHO PEN; good clinical pathways for early detection CVD, three priority cancers and asthma delivered through a multidisciplinary team (CHW, midwifes, family practitioners); coordinated care based on defined tasks with support of other disciplines relevant for NCD care (mental health, nutrition) and strong focus on self-care; CHW’s and midwives’ expanded scope of work with demonstrated pilot feasibility of IRA PEN model (good knowledge, skills to assess, advice and manage cardio-vascular risk and other NCD); good availability, affordability of essential medicines and technologies; and clear organizational structures from primary care centers to central level for oversight and support of IraPEN, with health Information management tools being available for programme monitoring at different levels^6^.

## Challenges & opportunities in the EMR

Perceived challenges include the uneven progress and needs across the WHO EM region; humanitarian emergencies and political instability; vertical approaches and programs; a lack of human and financial resources (both in ministries of health and WHO); and other health systems weaknesses such as poor capacity for guideline adaptation and development of tools; availability or affordability of NCD medicines; training and supportive supervision; NCD related data analysis; insufficient and unsustainable country support for NCD integration; and WHO PEN advocacy and communication.

Opportunities however exist through the global SDG and universal health coverage (UHC) agendas offering an opportunity to revisit essential health services package until 2030; a willingness to prioritize cost-effective interventions (e.g., WHO NCD Global Action Plan, Appendix 3, Update 2017) until 2020; the systematic NCD and CVD monitoring within the Global NCD Monitoring Framework until 2025; a better alignment with health sector reform and strengthening efforts and other integrated service delivery initiatives as well as the ‘One UN’ and ‘One WHO Integrated Country Support’ momentum e.g., UN NCD *interagency taskforce* and *investment case* missions^7^.

## Conclusions and recommendations

Overall, there has been strong political commitment to NCD governance, as evidenced by the EM Regional Committee’s endorsement of the regional framework for action. However, despite the clear roadmap, progress has been slow and scattered, differing vastly by country and by topic. The factors inhibiting progress include the complex emergencies affecting many countries in the region, in addition to economic constraints. Nevertheless, the WHO tools and frameworks have been well received across the region and are vital documents to support countries in the development, implementation and monitoring of their NCD policies, strategies and plans. The WHO EM regional office continues to provide technical support to its 22 countries, strongly supporting them in achieving the targets they have pledged to attain.

Specifically for CVD governance, national policies, strategies and plans, targets and indicators, stewardship, advocacy and information sharing should be further promoted. For CVD prevention and reduction of risk factors, the evidence-based NCD ‘best-buys’ for tackling tobacco use (i.e., WHO FCTC and MPOWER package) and alcohol abuse (where appropriate), unhealthy nutrition (excessive salt, *trans*-fatty acids, sugar intake) and physical inactivity have to be tackled more vigorously. In the area of CVD management and health care the full implementation of the recommendations of Global HEARTS initiative with a focus on treatment protocols at primary health care level should be propagated. Finally, in the field of CVD surveillance, monitoring and evaluation, countries are encouraged to regularly conduct NCD country capacity, STEP and other risk factor surveys, to generate more reliable cause-specific mortality data, and to agree on key performance indicators at facility level (making use of the Global HEARTS monitoring and evaluation module).

## References

[1] United Nations (UN) General Assembly (2011). Political Declaration on NCD.

[2] United Nations (UN) General Assembly (2014). Comprehensive Review and Outcome Document on NCD.

[3] United Nations (UN) (2016). Sustainable Development Goals (SDG): NCD targets and indicators.

[4] World Health Organization (WHO) (2012). Eastern Mediterranean Regional (EMR) Framework for Action on NCD.

[5] World Health Organization (WHO) (2017). Global Progress Monitor on NCD.

[6] (2017). The IraPEN experience in the Islamic Republic of Iran.

[7] United Nations (UN) (2017). NCD interagency taskforce and investment case missions.

